# Research hotspots and future trends of insomnia in Parkinson’s disease: a bibliometric and visualization analysis from 1973 to 2024

**DOI:** 10.3389/fnagi.2025.1535861

**Published:** 2025-05-09

**Authors:** Xianghong Guo, Tianye Wang, Dan Wang, Jingjing Zhou, Xin Lai

**Affiliations:** Suzhou Industrial Park Xinghai Hospital, Suzhou, Liaoning, China

**Keywords:** bibliometric analysis, Parkinson’s disease, insomnia, VOSviewer, CiteSpace

## Abstract

**Background and objectives:** Despite the growing body of research on Parkinson’s disease (PD) and insomnia, comprehensive analysis of overall research trends remains limited. This study aims to evaluate these trends and identify research hot spots using bibliometric analysis.

**Methods:**

Publications on PD and insomnia from 1973 to 2024 were extracted from the Web of Science Core Collection. Analytical tools such as VOSviewer, CiteSpace, and R 4.3.3 were employed for evaluation.

**Results:**

A total of 610 publications are analyzed, demonstrating a consistent upward trend. The United States leads the field with 150 articles, representing 24.59% of total output, followed by China with 79 publications. Harvard University is the top contributing institution with 44 articles. *Movement Disorders* ranks as the leading journal, publishing 28 papers (4.59% of the total) and also securing the top spot in total citations. The most prolific author is Lima Marcelo M. S., with 15 publications and 50 recorded collaborations. Burst keyword analysis revealed increasing interest in terms such as “validation,” “index,” and “scale” since 2019.

**Conclusion:**

Research on PD and insomnia exhibits a clear upward trend, reflecting increasing academic interest. Future studies are expected to focus on the validation of diagnostic tools, the development of scales, and the integration of artificial intelligence and personalized medicine for improved treatment precision.

## Introduction

Parkinson’s disease (PD) affects approximately 1% of individuals over 60 years of age (Tysnes and Storstein, 2017), and has a global prevalence surpassing 10 million people, making it the second most prevalent neurodegenerative disorder and a leading cause of neurological disability (Church, 2021). While PD is primarily characterized by motor symptoms such as bradykinesia, resting tremor, rigidity, and postural instability, which progressively impair daily functioning and quality of life, the burden of the disease extends far beyond these motor manifestations (Tolosa et al., 2021). PD is also associated with a wide array of non-motor symptoms (NMS), including sleep disturbances, hyposmia, constipation, urinary dysfunction, orthostatic hypotension, memory impairment, depression, and pain (Bloem et al., 2021). These NMS significantly complicate disease management and further degrade the quality of life in patients.

Among the non-motor symptoms, sleep disturbances are particularly prevalent, affecting up to 60% of PD patients (Mizrahi-Kliger et al., 2022). Insomnia, one of the most common forms of sleep disturbance, differs from other sleep disorders, such as REM sleep behavior disorder (RBD) or excessive daytime sleepiness (EDS), in that it is characterized by difficulty initiating or maintaining sleep, waking up too early, or experiencing non-restorative sleep, despite adequate sleep opportunity (Marinus et al., 2018). In PD, insomnia is uniquely multifactorial, often arising from a combination of motor symptoms—such as tremors, rigidity, or nocturnal akinesia—and non-motor symptoms, including nocturia, anxiety, depression, or medication side effects (Aarsland, 2016; Church, 2021). Unlike insomnia in the general population, Parkinson’s-related insomnia often coexists with fragmented sleep and other nocturnal disturbances, such as vivid dreams, periodic limb movements, or RBD, further exacerbating the complexity of sleep issues in these patients (Klingelhoefer et al., 2019). Its severity tends to worsen as the disease progresses, and it has been linked to poorer cognitive function, a heightened risk of mood disorders, and an increased burden of disease management (Assogna et al., 2020). Furthermore, insomnia in PD not only exacerbates motor symptoms but also contributes to emotional and psychological distress in patients, thereby complicating therapeutic interventions and worsening overall patient outcomes (Duan et al., 2025). These challenges also extend to caregivers, who frequently report impaired quality of life and increased psychological stress due to patients’ disrupted sleep patterns and associated behavioral changes (Broxson and Feliciano, 2020). Therefore, there is a pressing need for more precise and effective diagnostic tools and therapeutic approaches to mitigate the impact of insomnia on both patients and their families (Arias-Carrion et al., 2025).

Given the high prevalence and considerable impact of insomnia on the wellbeing of PD patients, it is crucial to explore the evolving research trends in this area. Recent years have seen a marked increase in the volume of scientific literature on PD-related insomnia, reflecting growing academic and clinical interest. However, the expansive scope of this body of research necessitates advanced analytical tools to effectively identify key trends and emerging insights. Bibliometric analysis offers a quantitative approach to evaluating the literature, enabling researchers to track the evolution of research, assess the impact of publications, and project future directions (Hassan and Duarte, 2024; Pritchard, 1969). This method allows for the visualization of collaborative networks, citation patterns, and research “hot spots,” and has been successfully applied in various fields to reveal pivotal research trends and innovations (Pei et al., 2022; Yarmohammadi et al., 2024). While several bibliometric studies on PD and other non-motor symptoms—such as sleep disorders, anxiety, and depression—have been conducted (Li et al., 2024; Sun et al., 2023; Zhang et al., 2023), there is a clear gap in bibliometric analyses specifically focused on insomnia in PD. Thus, this study aims to employ advanced bibliometric tools to comprehensively analyze the literature on PD and insomnia, providing a detailed understanding of the research landscape and exploring developmental trends in this important field.

## Materials and methods

### Search strategies and data collection

The literature search was performed using the Web of Science Core Collection (WoSCC), which offers comprehensive coverage of multiple disciplines across sciences, social sciences, and arts and humanities. In this study, the Science Citation Index Expanded (SCIE) and Social Science Citation Index (SSCI) were selected to obtain the most comprehensive and accurate studies investigating PD and insomnia. The search formula was as follows: [TS=̃ (Parkinson OR Parkinson’s Disease) AND TS = (insomnia OR sleep deprivation OR sleeplessness)]. To capture the most current data, the search was conducted within the timeframe from 1 January 1973, to 22 July 2024. To ensure consistency, the search was conducted on a single day (22 July 2024). Data were collected in text format, which included key information such as publication and citation counts, titles, author affiliations, institutions, countries/regions, keywords, and journal sources.

### Statistical analysis

Three robust bibliometric tools were utilized for visualization and comprehensive analysis: VOSviewer (version 1.6.20), CiteSpace (version 6.3.R1), and R (version 4.3.3). Data were extracted from bibliographic records and organized using Microsoft Excel. VOSviewer, a versatile software tool, was crucial for mapping collaborations, co-authorships, citations, and co-citations among institutions and authors (van Eck and Waltman, 2010). This software was utilized to perform country and institution analysis, journal and co-cited journal analysis, author and co-cited author analysis, as well as keyword co-occurrence analysis. CiteSpace is an information visualization software that creates visual knowledge graphs by analyzing citation relationships between documents, allowing researchers to intuitively grasp the development context and key nodes within a research field (Synnestvedt et al., 2005). In the visual analysis, the size of each node represents the number of associated publications, while the thickness of the lines connecting nodes reflects the strength of their relationships. Node colors may indicate different clusters or temporal aspects of the data. R 4.3.3 was utilized to export descriptive analysis results, a process also known as performance analysis within the bibliometric domain (Donthu et al., 2021).

To quantify the academic impact of individuals and journals, several metrics were utilized. The H-index, a key indicator for assessing a researcher’s academic contributions, offers insights into future performance. The G-index extends the H-index by evaluating citation distribution across a researcher’s publications, providing a more detailed view of citation impact (Borgohain et al., 2023). The M-index, derived by dividing the H-index by the number of years since a researcher’s first publication, assesses research performance over time. An M-index below 1 suggests average performance, between 1 and 2 indicates above-average performance, and values above 2 signify exceptional impact (Borgohain et al., 2023). Journal impact factor (IF), sourced from the Journal Citation Reports (JCR), provides a quantitative measure reflecting the average number of citations to articles published in journals, books, or symposium series (Górski et al., 2021).

## Results

### An overview of publications

**FIGURE 1:**
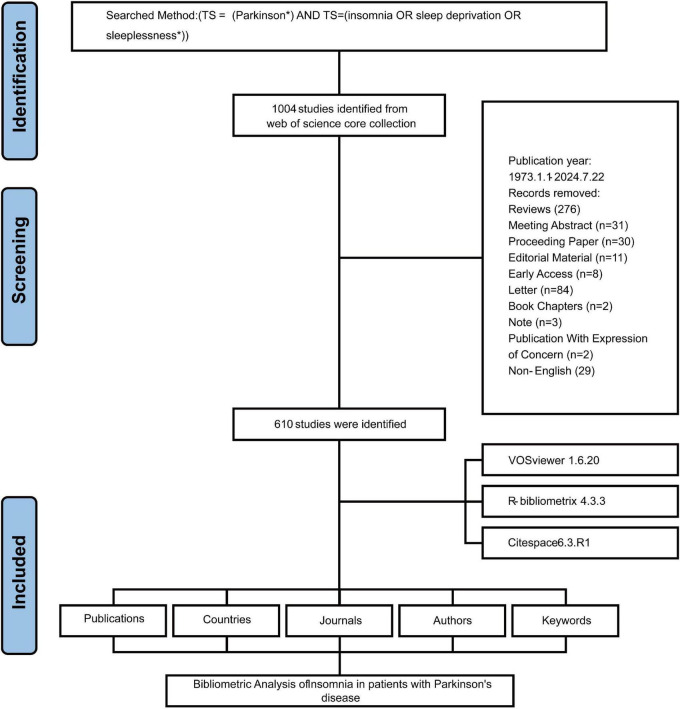
Flow chart.

**FIGURE 2:**
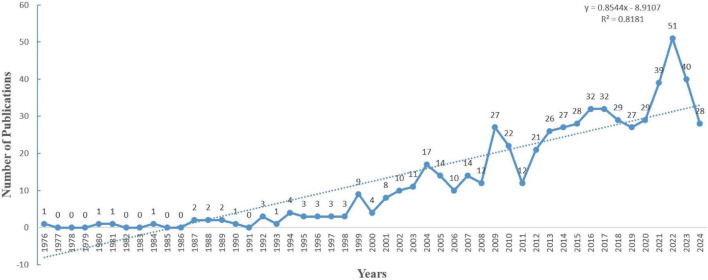
Annual publications from 1976 to 2024.

A total of 610 eligible publications were included in the final analysis, as outlined in the data screening process depicted in Figure 1. The publication trends from 1976 to 2024 can be divided into three distinct phases based on the volume of publications, reflecting an overall upward trajectory (Figure 2). The first phase, from 1976 to 1991, was characterized by minimal output, with an average of 0.69 articles per year. In the second phase (1992–2003), the publication rate increased significantly, averaging 6.20 articles per year, indicating a growing scholarly focus on the intersection of PD and insomnia. The third and most dynamic phase, spanning from 2004 to 2024, demonstrated fluctuating yet sustained growth, with an average of 25.57 articles per year, highlighting the expanding and evolving nature of research in this area.

The most cited article in this field is titled “6 months neurological and psychiatric outcomes in 236,379 survivors of COVID-19: a retrospective cohort study using electronic health records,” published in *The Lancet Psychiatry* (2023 IF = 30.8) in 2021, which has accumulated 1,116 citations (Taquet et al., 2021). The second most cited article is “The PRIAMO study: A multicenter assessment of non-motor symptoms and their impact on quality of life in Parkinson’s disease,” published in *Movement Disorders* (2023 IF = 7.4) in 2009, with 1,024 citations (Barone et al., 2009). The third most cited article is “The role of cerebrospinal fluid hypocretin measurement in the diagnosis of narcolepsy and other hypersomnias,” published in *Archives of Neurology* in 2002, which received 795 citations (Mignot et al., 2002). The top 20 most cited references are listed in Supplementary Table 1.

### Analysis of countries and institutions

**FIGURE 3:**
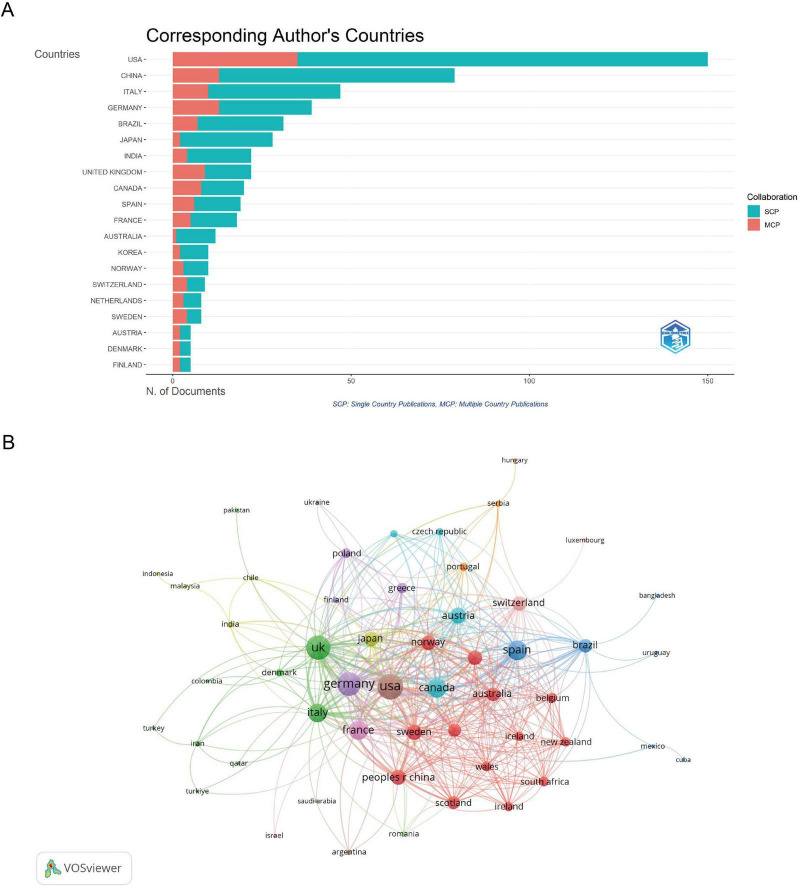
Information about the countries. **(A)** Distribution of corresponding author’s publications by country. SCP: Single Country Publications. MCP: Multiple Country Publications. **(B)** Visualization map depicting the collaboration among different countries. Nodes represent countries, with size indicating publication count. Links represent co-authorships, with thickness showing collaboration strength. Colors indicate different research clusters. Total link strength in collaboration networks measures the frequency of co-authorship between countries, indicating the level of collaborative research.

This analysis includes data from 60 countries and 135 institutions involved in PD and insomnia research. The distribution of corresponding authors’ publications, differentiating between single-country publications (SCP) and multiple-country publications (MCP), reflects the extent of international collaboration (Figure 3A). In terms of publication volume, the United States leads with 150 articles, accounting for 24.59% of the total research output. China follows with 79 publications, making it the second-largest contributor (Table 1). The United Kingdom stands out for having the highest average citations per article at 155.1, followed by Norway (105.1) and Canada (93.3). In terms of international collaboration, the co-authorship network (Figure 3B) demonstrates that the United States has the highest total link strength (165), indicating its prominent role in fostering global research partnerships. The United Kingdom (144) and Germany (136) also display strong collaborative networks (Table 2). Notably, smaller countries such as Norway and Switzerland, despite contributing fewer publications, have high average citation rates and strong collaborative ties.

**TABLE 1:** Publication and citation profiles of leading countries.

Country	Articles	Freq	MCP_Ratio	TP	TP_rank	TC	TC_rank	Average citations
United States	150	24.59	23.33	632	1	8179	1	54.5
China	79	12.95	16.46	322	2	946	7	12
Italy	47	7.70	21.28	261	3	2709	3	57.6
Germany	39	6.39	33.33	207	3	1580	5	40.5
Brazil	31	5.08	22.58	112	3	816	9	26.3
Japan	28	4.59	7.14	107	3	374	15	13.4
India	22	3.61	18.18	45	3	481	14	21.9
United Kingdom	22	3.61	40.91	161	3	3413	2	155.1
Canada	20	3.28	40.00	96	3	1866	4	93.3
Spain	19	3.11	31.58	117	3	506	13	26.6
France	18	2.95	27.78	132	3	568	11	31.6
Australia	12	1.97	8.33	73	3	817	8	68.1
Korea	10	1.64	20.00	41	3	116	21	11.6
Norway	10	1.64	30.00	56	3	1051	6	105.1
Switzerland	9	1.48	44.44	32	3	287	18	31.9
Netherlands	8	1.31	37.5	68	3	511	12	63.9
Sweden	8	1.31	50	38	3	367	16	45.9
Austria	5	0.82	40	29	3	154	19	30.8
Denmark	5	0.82	40	18	3	124	20	24.8
Finland	5	0.82	40	21	3	94	25	18.8

Articles, publications of corresponding authors only; Freq, frequence of total publications; MCP_Ratio, proportion of multiple country publications; TP, total publications; TP_rank: rank of total publications; TC, total citations; TC_rank, rank of total citations; Average citations, the average number of citations per publication.

**TABLE 2:** Collaboration analysis.

Rank	Country	Documents	Citations	Total link strength
1	United States	178	11,293	165
2	United Kingdom	59	5,801	144
3	Germany	65	3,468	136
4	Spain	36	2,046	95
5	France	37	2,716	89
6	Canada	31	2,788	88
7	Italy	62	3,989	86
8	Austria	15	1,814	62
9	China	90	1,841	57
10	Sweden	17	1,103	55
11	Norway	14	2,395	53
12	Switzerland	15	1,614	52
13	Japan	35	1,551	50
14	Netherlands	17	2,256	50
15	Australia	20	1,919	46
16	Brazil	40	2,015	46
17	Scotland	8	671	41
18	Singapore	6	571	40
19	Greece	8	363	27
20	Belgium	4	475	24

**FIGURE 4:**
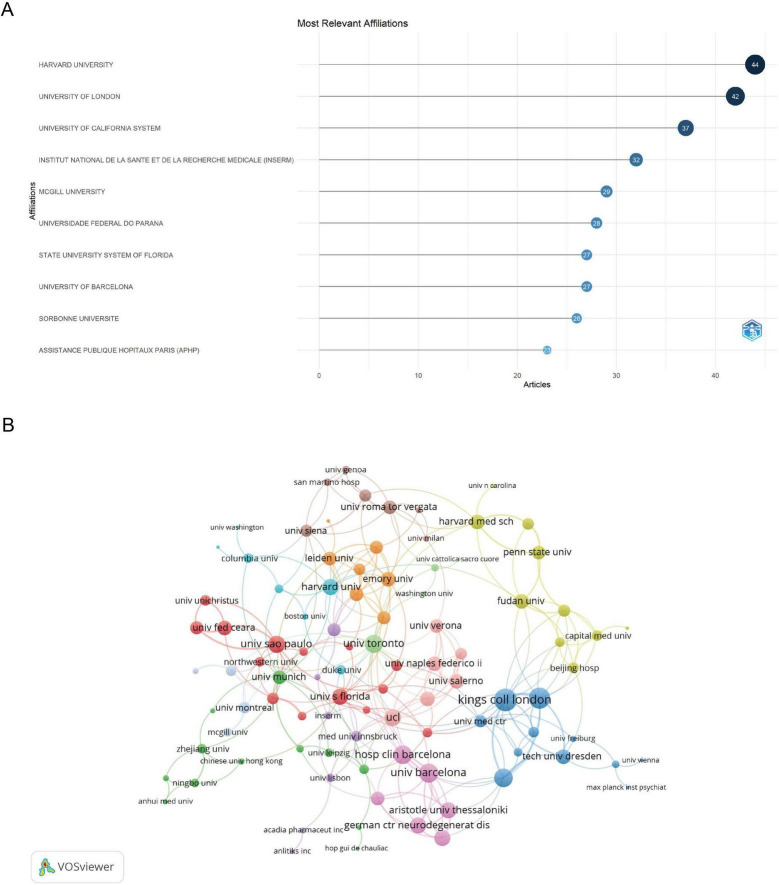
Information about the institutions. **(A)** Top ten institutions by article count and rank. Circle size shows article count. Darker shades indicate higher ranks. **(B)** Visualization map depicting the collaboration among different institutions. Nodes represent institutions, with size indicating publication count. Links represent co-authorships, with thickness showing collaboration strength. Colors indicate different research clusters. Total link strength in collaboration networks measures the frequency of co-authorship between institutions, indicating the level of collaborative research.

The analysis of institutions involved in PD and insomnia research shows that the top ten institutions contributed 315 publications (Figure 4A), representing 51.64% of the total research output. Harvard University and the University of London are the leading contributors, with 44 and 42 articles, respectively (Figure 4A). Other major contributors include the University of California system (37 articles), INSERM (32 articles), and McGill University (29 articles). In terms of collaboration, several institutions display strong international ties. King’s College London and King’s College Hospital London exhibit the highest total link strengths, at 25 and 22, respectively, indicating frequent co-authorship with other institutions (Figure 4B and Supplementary Table 1). Other key collaborators include the University of Barcelona (link strength 19), Carlos III Health Institute (Zhang et al., 2023), and the University of Toronto (Sun et al., 2023). The collaboration network shows clusters where institutions like King’s College London, the University of Barcelona, and Harvard University form strong hubs of research activity.

### Analysis of journals

**FIGURE 5:**
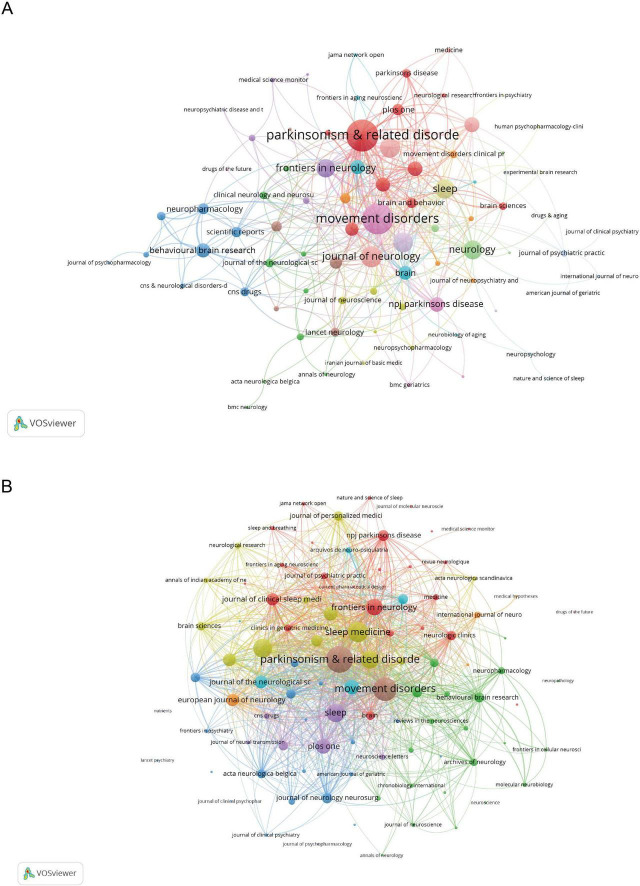
Information about the Journals. **(A)** Distribution of publications in different journals. **(B)** The network map of co-cited journals. Nodes represent journals, with size indicating publication count. Links represent co-authorships, with thickness showing collaboration strength. Colors indicate different research clusters. Total link strength in collaboration networks measures the frequency of co-authorship between countries, indicating the level of collaborative research.

Of the top 20 journals, 15 are based in Europe (Table 3). The Movement Disorders leads with 28 papers, accounting for 4.59% of the total, followed by Parkinsonism and Related Disorders with 25 papers and Sleep with 18 papers. Meanwhile, Movement Disorders also ranks first in total citations. Although Lancet Neurology is 17th in publication volume with six papers, it boasts the highest impact factor of 46.5 in 2023 and has the most total citations. Furthermore, the journal co-occurrence network comprises 97 journals, each appearing at least twice (Figure 5A). The top three journals by total link strength are Parkinsonism and Related Disorders (112), Movement Disorders (108), and Journal of Neurology (56). In the journal coupling network, which includes 119 journals with at least five pairs of couplings (Figure 5B), Parkinsonism and Related Disorders leads with a total link strength of 3,874, followed by Movement Disorders (3,125) and Sleep Medicine (2,476).

**TABLE 3:** Bibliometric indicators of high-impact journals.

Journal	H_index	IF	JCR_ Quartile	PY_start	TP	TP_rank	TC	TC_rank
Movement Disorders	20	7.4	Q1	2001	28	1	1816	1
Parkinsonism & Related Disorders	17	3.1	Q2	2000	25	2	479	6
Sleep	15	5.3	Q1	1997	18	3	930	3
Journal of Neurology	13	4.8	Q1	1984	15	5	312	9
Neurology	12	7.7	Q1	1981	12	7	1570	2
Sleep Medicine	11	3.8	Q1	2005	17	4	650	4
Behavioural Brain Research	8	2.6	Q3	2004	10	8	149	30
Plos One	8	2.9	Q1	2011	10	10	255	12
Frontiers in Neurology	7	2.7	Q3	2016	13	6	69	66
Journal of Parkinson’s Disease	7	4	Q2	2013	10	9	91	50
European Journal of Neurology	6	4.5	Q1	1998	8	12	156	28
Journal of Clinical Sleep Medicine	6	3.5	Q1	2012	7	13	173	26
Lancet Neurology	6	46.5	Q1	2010	6	17	244	15
Neurological Sciences	6	2.7	Q3	2000	9	11	113	40
Scientific Reports	6	3.8	Q1	2014	7	14	60	79
Archives of Neurology	5	#N/A	#N/A	2002	5	20	#N/A	#N/A
CNS Drugs	5	7.4	Q1	1999	5	21	54	86
Journal of Neurology Neurosurgery and Psychiatry	5	8.7	Q1	1990	5	22	552	5
Journal of Sleep Research	5	3.4	Q2	1994	5	23	213	20
Journal of the Neurological Sciences	5	3.6	Q2	2004	6	16	231	17

H_index, the h-index of the journal, which measures both the productivity and citation impact of the publications; IF, impact factor, indicating the average number of citations to recent articles published in the journal; JCR_Quartile, the quartile ranking of the journal in the journal citation reports, indicating the journal’s ranking relative to others in the same field (Q1: top 25%, Q2: 25%–50%, Q3: 50%–75%, Q4: bottom 25%); TP, total publications; TP_rank, rank of total publications; TC, total citations; TC_rank, rank of total citations; Average citations; the average number of citations per publication; PY_start; publication year start, indicating the year the journal started publication.

### Analysis of authors

**FIGURE 6:**
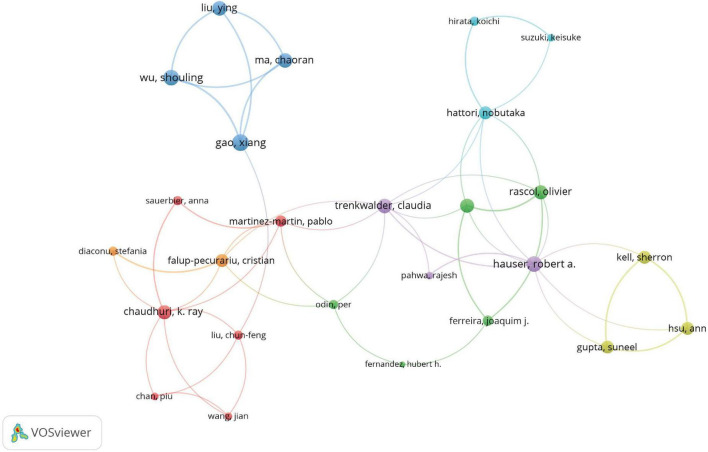
Information about the authors. Visualization map depicting the collaboration among different authors. Nodes represent authors, with size indicating publication count. Links represent co-authorships, with thickness showing collaboration strength. Colors indicate different research clusters. Total link strength in collaboration networks measures the frequency of co-authorship between authors, indicating the level of collaborative research.

The contributions of 3,918 authors were noted, with 28 articles (4.59%) authored by a single individual. This indicates that the majority of articles involved multiple authors, with an average of 7.33 authors per publication. Notably, international collaboration is prevalent, with transnationally co-authored articles comprising 25.25% of the total (Table 4). Among the 104 authors with over three international collaborations, Lima Marcelo M. S. stands out with 50 collaborations (Figure 6 and Supplementary Table 2). He is followed by Targa Adriano D. S. with 41 collaborations and Rodrigues Lais S. with 40, reflecting their significant global engagement in the field.

**TABLE 4:** Publication and citation profiles of high-impact authors.

Authors	H_index	G-index	M-index	PY_start	TP	TP_Frac	TP_rank	TC	TC_rank
Lima Marcelo M. S.	10	15	0.588	2008	15	2.121	1	73	1
Arnulf Isabelle	6	7	0.353	2008	7	1.233	4	14	100
Chaudhuri K. Ray	6	7	0.400	2010	7	0.795	5	17	68
Da Cunha Claudio	6	6	0.500	2013	6	0.817	8	38	3
Hauser Robert A.	6	6	0.333	2007	6	0.744	10	5	366
Noseda Ana Carolina D.	6	7	0.545	2014	7	0.959	6	29	42
Rodrigues Lais S.	6	10	0.545	2014	10	1.395	3	31	39
Targa Adriano D. S.	6	11	0.545	2014	11	1.538	2	31	40
Willis Gregory L.	6	6	0.333	2007	6	2.167	12	19	54
Barone Paolo	5	5	0.313	2009	5	0.376	14	39	2
POEWE WERNER	5	5	0.417	2013	5	0.726	17	3	585
Postuma Ronald B.	5	6	0.455	2014	6	1.010	11	18	64
Trenkwalder ClaudiA	5	5	0.313	2009	5	0.409	19	14	113
Andersen Monica L.	4	4	0.235	2008	4	0.542	20	31	38
Antonini Angelo	4	4	0.250	2009	4	0.228	21	37	5
Aurich Marian A. F.	4	5	0.364	2014	5	0.835	13	24	47
Cortelli Pietro	4	6	0.250	2009	6	0.683	7	20	51
Dauvilliers Yves	4	4	0.364	2014	4	0.325	25	13	116
Erro Roberto	4	4	0.308	2012	4	0.343	27	6	322
Ferraz Anete C.	4	4	0.308	2012	4	0.549	29	33	19

H_index, the H-index of the journal, which measures both the productivity and citation impact of the publications; G_index, the g-index of the journal, which gives more weight to highly-cited articles; M_index: the m-index of the journal, which is the h-index divided by the number of years since the first published paper; TP, total publications; TP_rank, rank of total publications; TC, total citations; TC_rank, rank of total citations; Average citations, the average number of citations per publication; PY_start, publication year start, indicating the year the journal started publication.

### Analysis of keywords

**FIGURE 7:**
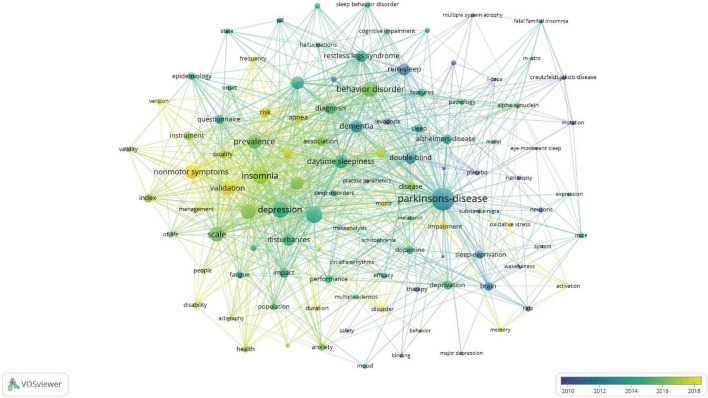
Visual analysis of keyword co-occurrence network analysis. This network visualization displays the co-occurrence of keywords in selected literature. Each node represents a keyword, with size indicating its frequency of occurrence. Links between nodes represent co-occurrence in the same documents, with thicker lines showing stronger associations. Colors reflect the average publication year of the articles, as indicated by the color gradient at the bottom right.

The keyword analysis identified 1,290 distinct keywords. To uncover research hotspots and emerging trends, a co-occurrence network of keywords was generated (Figure 7). The largest node, “Parkinson’s disease,” is the dominant keyword, reflecting its central role in this body of research. Other frequently occurring keywords include “insomnia,” “depression,” and “prevalence.” Keywords that emerged early in the research may be highlighted in blue, while more recently appearing terms may be marked in yellow. Terms such as “validation,” “scale,” and “risk” indicate a shift toward assessing diagnostic tools. Additionally, the presence of keywords like “sleep deprivation” and “disturbances” reflects the increasing recognition of sleep-related issues as critical components of PD research. The keywords such as “dopamine,” “melatonin,” and “therapy” suggest a continued focus on understanding the underlying pathophysiology of PD and developing therapeutic interventions.

**FIGURE 8:**
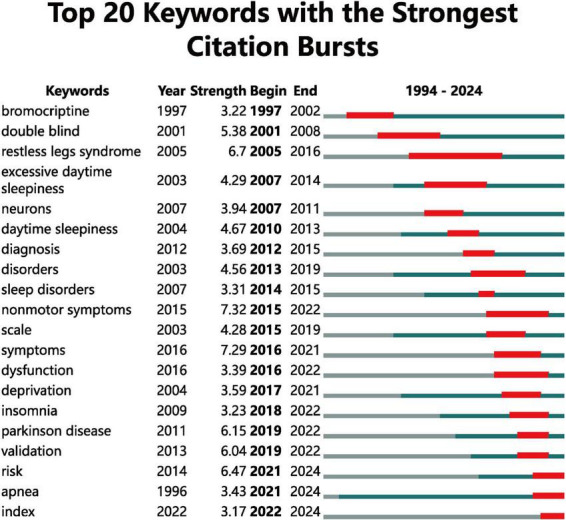
Top 20 Keywords with the Strongest Citation Bursts. The dark blue line indicates keywords with a consistent citation frequency, while the red line represents keywords that have experienced a substantial increase in frequency, signifying high popularity.

The analysis of keywords with significant citation bursts provides valuable insights into evolving research trends from 1994 to 2024 (Figure 8). One of the earliest keywords to experience a strong citation burst was “bromocriptine,” which saw heightened attention from 1997 to 2002, reflecting its early significance in PD research. “Symptoms” and “dysfunction” have shown strong bursts from 2016 to 2022, further indicating the field’s focus on the broader clinical spectrum of PD beyond motor impairments. Emerging research areas are also evident in the bursts for keywords such as “risk,” “apnea,” and “index,” which began in 2021 and are projected to continue through 2024. These keywords suggest a growing interest in risk assessment, sleep apnea, and the development of new indices or metrics for evaluating patient outcomes. Additionally, “validation” of clinical tools and “scale” development have been prominent from 2019 to 2022, reflecting efforts to refine measurement instruments for PD and insomnia-related symptoms.

## Discussion

### General summary

This study provides a comprehensive bibliometric analysis of the research landscape on PD and insomnia from 1976 to 2024. A total of 610 publications were analyzed, revealing an increasing trend. Research output has steadily increased over the years, with the United States and China emerging as the leading contributors, while the United Kingdom and Norway stand out for their high citation impact. Harvard University and the University of London were identified as the top institutions, and Lima Marcelo M. S. was the most prolific author. Frequently cited journals include *Movement Disorders*, *Lancet Neurology*, and *Parkinsonism & Related Disorders*, while prominent keywords such as “Parkinson’s disease,” “insomnia,” “machine learning,” and “non-motor symptoms” highlight the evolving focus of this field. Emerging trends suggest a growing interest in advanced diagnostic/predictive tools and non-motor symptoms.

The most cited article highlights the growing interest in the relationship between systemic illnesses, such as COVID-19, and neurological or psychiatric outcomes, including insomnia. Its findings underscore the potential role of neuroinflammation and systemic factors in exacerbating non-motor symptoms in neurodegenerative diseases like PD (Taquet et al., 2021). While not specific to PD, this study broadens the understanding of how sleep disturbances might arise or worsen in the context of broader neurological conditions, offering valuable insights for related research fields. Similarly, the second most cited article, the PRIAMO study, represents a pivotal advancement in the understanding of NMS in PD. By systematically demonstrating the prevalence and significant impact of NMS—including insomnia—on patients’ quality of life, this study has reshaped clinical priorities in PD care (Barone et al., 2009). It emphasizes the need for a multidisciplinary approach, addressing sleep disturbances and other NMS alongside traditional motor symptom management. Lastly, the third most cited article introduces cerebrospinal fluid (CSF) hypocretin measurement as a biomarker for narcolepsy, establishing a critical link between hypocretin dysregulation and sleep disorders (Mignot et al., 2002). Although its primary focus is narcolepsy and hypersomnias, this study is highly relevant to PD research, as emerging evidence suggests that hypocretin pathways might also contribute to sleep disturbances in PD. Together, these high quality studies highlight the interconnectedness of systemic, neurodegenerative, and sleep-related mechanisms, offering essential insights into the management of insomnia in PD and related conditions.

The analysis of countries and institutions demonstrates the dominant role of the United States and China in PD and insomnia research. The United States leads in publication volume, likely due to its strong funding infrastructure for neurodegenerative research, supported by agencies such as the National Institutes of Health (NIH) and private foundations like the Michael J. Fox Foundation. Institutions such as Harvard University and the University of California system have long been at the forefront of PD research, reflecting both their clinical and basic research expertise. These institutions also benefit from international collaborations with key partners like King’s College London, which further enhance their global reach and research output. The complexity of PD, which involves both motor and non-motor symptoms such as insomnia, drives the need for a multidisciplinary research approach involving clinical neurology, psychiatry, neuroscience, and pharmacology (Kalia and Lang, 2015).

China has rapidly increased its research output in recent years, reflecting the country’s growing investment in biomedical research and drug development, supported by initiatives such as the National Natural Science Foundation of China (NSFC). While China contributes significantly to the publication volume, the relatively lower average citation rate compared to Western countries suggests that Chinese research is still in the process of gaining broader international recognition. Factors such as the quality of research, language barriers, or limited early-stage international collaborations may contribute to this gap (Li et al., 2022). Smaller countries like Norway and Switzerland, despite contributing fewer publications, show high average citation rates and strong collaborative networks. These countries have focused on specialized areas, such as advanced imaging techniques (e.g., MRI) and machine learning applications for PD diagnosis, which likely explains their high impact despite limited output. Their collaborations with larger institutions further amplify their contributions to the field.

A core finding from the journal analysis is that *Movement Disorders* and *Parkinsonism & Related Disorders* are the leading journals in this field, both in terms of publication count and citation impact. These journals primarily focus on neurodegenerative diseases, with a strong emphasis on the clinical and pathological aspects of PD, which makes them natural hubs for research on non-motor symptoms such as insomnia. Their high citation rates reflect the relevance and quality of the research they publish, especially studies addressing the complex interplay between PD and sleep disturbances. Additionally, *Lancet Neurology*, despite publishing fewer articles in this domain, stands out with the highest impact factor. For example, one study published in *Lancet Neurology* focused on the role of cerebrospinal fluid hypocretin measurement in diagnosing narcolepsy and hypersomnias, providing insights into how similar biomarkers could be applied to sleep disorders in PD (Bourgin et al., 2008). Another study explored the neurological outcomes of PD patients following experimental treatments using advanced imaging and sleep monitoring techniques (Soileau et al., 2022). These findings highlight the journal’s preference for cutting-edge research with clinical implications, such as novel diagnostic tools or treatments for sleep disorders in PD patients.

The prominence of European journals in this field, particularly those based in the United Kingdom and Germany, reflects the historical and ongoing commitment of European researchers to neurodegenerative disease research. Journals like *Sleep Medicine* and *Journal of Neurology*, which are also central to this field, further underscore the interdisciplinary nature of PD research, as they cater to both neurological and sleep-related studies.

The author analysis highlights Lima Marcelo M. S. as the most prolific contributor to the field. His research has primarily focused on non-motor symptoms of PD, particularly sleep disturbances, contributing to a better understanding of how these symptoms affect disease progression and patient quality of life (Lima et al., 2012). His collaboration with other leading researchers, such as Targa Adriano D. S. and Rodrigues Lais S., has also helped advance the understanding of non-motor symptoms associated with PD globally. The high average number of co-authors per article reflects the interdisciplinary nature of PD research, which often requires expertise spanning neurology, sleep medicine, psychology, and biomedical engineering. This trend underscores the importance of global research networks, as PD’s complex symptoms call for a multifaceted approach to clinical management and research. The prevalence of international collaborations highlights the importance of pooling expertise from different countries to tackle the multifactorial nature of PD (Feigin et al., 2021).

### Research hotspots and trends

The keyword analysis reveals several important research hotspots and trends. Unsurprisingly, terms like “Parkinson’s disease” and “insomnia” are the most frequently occurring keywords, reflecting the core focus of the analyzed literature. However, newer keywords such as “machine learning,” “deep learning,” and “MRI” suggest that researchers are increasingly interested in leveraging artificial intelligence (AI) and advanced imaging techniques to improve the diagnosis and treatment of PD and its associated sleep disturbances. Recent studies have demonstrated that AI-driven technologies, such as machine learning algorithms, can analyze large datasets—including genetic, imaging, and clinical data—to improve diagnostic accuracy and predict disease progression (Alowais et al., 2023). For example, deep learning models based on MRI scans have shown promise in detecting early-stage PD by identifying subtle changes in brain structure that are often associated with motor and non-motor symptoms, including insomnia (Garcia Santa Cruz et al., 2023). Other AI tools have been used to predict sleep disturbances in PD patients by analyzing polysomnography data and wearable device outputs, offering an innovative approach to personalized medicine (Zhang et al., 2020).

In terms of treatment, AI-driven approaches are being applied to optimize therapy regimens for PD patients. For example, recent research has used predictive algorithms to analyze patient responses to sleep medications and adjust dosages or combinations based on individual profiles (Yue et al., 2024). These advancements have the potential to improve not only symptom management but also long-term outcomes for patients with PD-related insomnia. However, while these technologies are promising, their integration into routine clinical practice remains limited by challenges such as cost, accessibility, and the need for further validation in diverse patient populations.

These technologies are being used to analyze large datasets, such as MRI scans, to enhance early diagnosis and predict disease progression, which could lead to earlier and more personalized interventions. For instance, a recent study highlighted the use of AI in predicting therapeutic outcomes for PD patients suffering from sleep disorders, demonstrating that machine learning models could distinguish patients who are likely to benefit from specific therapies, such as melatonin or dopamine agonists, from those who are not (Gupta et al., 2023). Such findings underline the growing role of AI in improving treatment efficacy and reducing trial-and-error approaches in clinical settings. Moreover, the increasing focus on validation tools is evident in recent studies that aim to standardize diagnostic scales for sleep disturbances in PD, allowing for more precise assessments of insomnia severity and its impact on quality of life (Yang et al., 2022). These efforts directly address the need for more reliable diagnostic tools, which is a critical step toward advancing precision medicine in this field. While AI and precision medicine have shown great potential, their clinical application is still in its infancy. Future research should focus on addressing current limitations, such as the lack of large-scale, multicenter validation studies, and on expanding access to these technologies in low-resource settings. Additionally, cross-disciplinary collaboration between neurologists, sleep specialists, and AI experts will be key to translating these innovations into widespread clinical use.

Another prominent trend is the increasing focus on non-motor symptoms, such as depression, dementia, and excessive daytime sleepiness, which have gained more attention in recent years due to their significant impact on patients’ quality of life. The strong citation bursts for keywords like “non-motor symptoms” and “daytime sleepiness” in the last decade indicate growing recognition of the importance of addressing these symptoms alongside the traditional motor impairments of PD. This shift reflects a broader trend toward a more holistic understanding of PD that considers the full spectrum of its impact on patients (Salawu et al., 2010).

### Keyword bursts and research evolution

The analysis of keyword bursts over time reveals key shifts in research focus. For instance, in earlier years, treatment-related terms such as “levodopa” or “dopamine agonists” were more prominent, reflecting the focus on pharmacological management of PD’s motor symptoms. More recent bursts, such as those for “machine learning” and “deep learning,” reflect the growing interest in utilizing artificial intelligence (AI) for both diagnostic and therapeutic advancements in PD. For example, a study by Noor et al. demonstrated how deep learning models could analyze MRI images to detect early signs of PD, with an emphasis on non-motor symptoms like insomnia (Noor et al., 2020). Another study applied machine learning algorithms to assess the effectiveness of sleep therapies in PD patients, showing promise for personalized treatment approaches (Eskofier et al., 2016). These trends suggest that the field is moving toward the integration of digital technologies to enhance precision in both diagnosis and treatment strategies.

The burst in keywords like “non-motor symptoms” and “sleep disturbances” during the last decade indicates that these research areas have become more central to understanding the overall impact of PD on patients. This shift is likely driven by increasing evidence that non-motor symptoms, particularly sleep disorders, significantly impair the quality of life and may even accelerate disease progression (Huang et al., 2019). Additionally, the emergence of keywords like “cancer” and “radiomics” may reflect growing interest in the relationship between PD and other chronic conditions (Schernhammer et al., 2006). Some studies have suggested that PD patients may have an altered risk of developing certain cancers, such as breast cancer, which could open new avenues for research into shared pathophysiological mechanisms (Schapira et al., 2017).

### Advantages and shortcomings

This study offers several advantages. First, it is the first to apply bibliometric methods specifically to Parkinson’s disease (PD) and insomnia research, providing valuable guidance for scholars and clinicians. The analysis identifies research hotspots and trends that align with recent clinical advancements, such as the integration of artificial intelligence in diagnosing and treating PD-related insomnia. The observed increase in studies on machine learning and non-motor symptoms reflects growing interest in personalized medicine and early diagnostics, which are critical for improving patient outcomes. These findings highlight areas where future research could bridge gaps, such as optimizing treatment regimens for insomnia in PD patients.

By using three bibliometric tools, including VOSviewer and CiteSpace, enhances the objectivity of the data analysis. Additionally, bibliometric analysis offers deeper insights into research hotspots and frontiers compared to traditional reviews. The exclusive focus on original research articles ensures that the findings reflect trends in primary research. However, this approach may limit the scope by excluding reviews or other publication types, which could provide broader context. Future studies may consider incorporating other publication types to complement the trends identified here.

This study also has limitations. It relies solely on the WoSCC database, potentially missing relevant studies from databases like Scopus or PubMed. While Web of Science is widely used in bibliometric analysis due to its comprehensive coverage of high-impact journals and robust citation data (Singh et al., 2021), relying on a single database introduces selection bias. Additionally, the exclusion of non-English publications may underestimate research conducted in other languages. Additionally, filtering out non-English publications may lead to an underestimation of research in those languages. Furthermore, the study did not evaluate the quality of included research beyond publication and citation counts. Bibliometric analysis excels at identifying trends but does not account for methodological rigor or clinical impact. For instance, randomized controlled trials (RCTs), which provide high-quality evidence, were not differentiated from observational studies. Future research could incorporate metrics for study design and quality to provide a more nuanced understanding of the research landscape. Lastly, bibliometric analysis highlights trends and research hotspots but does not capture longitudinal insights into clinical impacts. For example, while advancements in AI show promise, their actual contributions to improving diagnostic accuracy and treatment efficacy for PD-related insomnia remain unclear. Future longitudinal studies are needed to evaluate these outcomes and ensure emerging technologies fulfill their potential to improve patient care. Also, while bibliometric trends highlight an increasing focus on validation methods, there is still a gap in translating these advancements into standardized clinical guidelines. We emphasize the need for prospective studies and multicenter trials to assess the real-world effectiveness of these methods.

## Conclusion

Research on PD and insomnia holds significant value and potential. The growing number of papers reflects its increasing importance to scholars globally. The United States and China are leading in this field, though there’s a need for enhanced international collaboration. Besides basic research, emphasis should also be placed on translating research findings into practical applications.

## Data Availability

The original contributions presented in this study are included in this article/Supplementary material, further inquiries can be directed to the corresponding author.
