# Supplementary material for: Research hotspots and future trends of insomnia in Parkinson’s disease: a bibliometric and visualization analysis from 1973 to 2024
Source: Front Aging Neurosci. 2025 May 9;17:1535861. doi: 10.3389/fnagi.2025.1535861 (PMC12098332; doi:10.3389/fnagi.2025.1535861)
Supplement: Supplementary file 1 [file Table_1.docx]

Supplementary Table 1. Institutions Ranking by Number of Articles Published

| **Id** | **Organization** | **Documents** | **Citations** | **Total Link Strength** |
| --- | --- | --- | --- | --- |
| 469 | Kings Coll London | 10 | 294 | 25 |
| 467 | Kings Coll Hosp London | 7 | 127 | 22 |
| 876 | Univ Barcelona | 10 | 334 | 19 |
| 106 | Carlos Iii Inst Hlth | 6 | 222 | 18 |
| 346 | Hosp Clin Barcelona | 6 | 397 | 18 |
| 1085 | Univ Toronto | 6 | 353 | 17 |
| 1068 | Univ Sao Paulo | 8 | 238 | 16 |
| 859 | Ucl | 8 | 1056 | 15 |
| 35 | Aristotle Univ Thessaloniki | 3 | 213 | 14 |
| 277 | German Ctr Neurodegenerat Dis Dzne | 3 | 213 | 14 |
| 312 | Harvard Univ | 5 | 332 | 14 |
| 1004 | Univ Med Sch | 3 | 213 | 14 |
| 1061 | Univ S Florida | 9 | 674 | 14 |
| 237 | Emory Univ | 6 | 248 | 12 |
| 269 | Fudan Univ | 5 | 69 | 12 |
| 311 | Harvard Med Sch | 6 | 135 | 12 |
| 826 | Tech Univ Dresden | 6 | 161 | 12 |
| 919 | Univ Fed Ceara | 7 | 313 | 12 |
| 987 | Univ London Imperial Coll Sci Technol & Med | 5 | 834 | 12 |
| 1021 | Univ Naples Federico Ii | 4 | 158 | 12 |
