# Supplementary material for: Research hotspots and future trends of insomnia in Parkinson’s disease: a bibliometric and visualization analysis from 1973 to 2024
Source: Front Aging Neurosci. 2025 May 9;17:1535861. doi: 10.3389/fnagi.2025.1535861 (PMC12098332; doi:10.3389/fnagi.2025.1535861)
Supplement: Supplementary file 2 [file Table_2.docx]

Supplementary Table 2. Top 20 Researchers Ranked by Collaboration Strength

| **id** | **author** | **documents** | **citations** | **total link strength** |
| --- | --- | --- | --- | --- |
| 1566 | Lima Marcelo M. S. | 15 | 263 | 50 |
| 2706 | Targa Adriano D. S. | 11 | 143 | 41 |
| 2289 | Rodrigues Lais S. | 10 | 141 | 40 |
| 1964 | Noseda Ana Carolina D. | 7 | 132 | 32 |
| 504 | Da Cunha Claudio | 6 | 150 | 26 |
| 102 | Aurich Mariana F. | 5 | 89 | 24 |
| 656 | Eckeli Alan Luiz | 4 | 82 | 21 |
| 2096 | Pena-Pereira Marcio Alexandre | 4 | 82 | 21 |
| 2558 | Sobreira-Neto Manoel Alves | 4 | 82 | 21 |
| 2792 | Tumas Vitor | 4 | 82 | 21 |
| 785 | Franca Fernandes Regina Maria | 3 | 74 | 18 |
| 1952 | Nisihara Chagas Marcos Hortes | 3 | 74 | 18 |
| 2709 | Tavares Sobreira Emmanuelle Silva | 3 | 74 | 18 |
| 591 | Diaz-Lucena Daniela | 3 | 213 | 17 |
| 1252 | Kanata Eirini | 3 | 213 | 17 |
| 1625 | Llorens Franc | 3 | 213 | 17 |
| 2426 | Schmitz Matthias | 3 | 213 | 17 |
| 2543 | Sklaviadis Theodoros | 3 | 213 | 17 |
| 3167 | Zerr Inga | 3 | 213 | 17 |
| 56 | Andersen Monica L. | 4 | 95 | 15 |
